# Perceived usefulness of receiving a potential smoking cessation intervention via mobile phones among smokers in Indonesia

**DOI:** 10.12688/wellcomeopenres.15135.2

**Published:** 2020-08-19

**Authors:** Mochammad Fadjar Wibowo, Anil A. Kumar, Surahyo Sumarsono, Rashmi Rodrigues

**Affiliations:** 1 Faculty of Medicine, Public Health and Nursing, Universitas Gadjah Mada, Yogyakarta, Indonesia; 2 St John’s National Academy of Health Sciences, Bangalore, India; 3 Karolinska Institutet, Stockholm, Sweden; 4 Wellcome Trust/DBT India Alliance, New Delhi, India

**Keywords:** mHealth, mobile phones, smoking cessation, Indonesia

## Abstract

**Background:** The use of technology to support healthcare in Indonesia holds new promise in light of decreasing costs of owning mobile devices and ease of access to internet. However, it is necessary to assess end-user perceptions regarding mobile health interventions prior to its implementation. This would throw light on the acceptability of mobile phone communication in bringing about behavioral changes among the target Indonesian population. The aim of this study was to explore the perceived usefulness of receiving a potential smoking cessation intervention via mobile phones.

**Methods:** This is an exploratory cross-sectional study involving current and former adult tobacco smokers residing in Indonesia. Online advertisement and snowballing were used to recruit respondents. Data was collected using a web-based survey over a period of 4 weeks. Those willing to participate signed an online consent and were subsequently directed to the online questionnaire that obtained demographics, tobacco usage patterns, perceived usefulness of a mobile phone smoking cessation application and its design.

**Results:** A total of 161 people who smoked tobacco responded to the online survey. The mean age of the participants was 29.4. Of the 123 respondents, 102 were men. Prior experience with using a mobile phone for health communication (OR 3.6, *P*=0.014) and those willing to quit smoking (OR 5.1, *P*=0.043) were likely to perceive a mobile phone smoking cessation intervention as useful. A smartphone application was preferred over text messages, media messages or interactive voice response technology. Content consisting of motivational messages highlighting the methods and benefits of quitting smoking were requested.

**Conclusion:** People who smoke in Indonesia perceived receiving a potential smoking cessation intervention via mobile phones as useful. A multi-component, personalized smartphone application was the desired intervention technique. Such an intervention developed and implemented within a public health program could help address the tobacco epidemic in Indonesia.

## Introduction

With an estimated population of 266 million people in the year 2018, Indonesia is the fourth most populous country in the world ^1^ and the third highest number of tobacco smokers. Approximately 65% of adult males in Indonesia smoke tobacco ^2^. Of those who smoke tobacco, 61% start smoking before the age of 19 years ^3^ with a 19% prevalence of smoking among Indonesian teenagers aged 13–15 years ^3^.

The large revenue from cigarettes despite low taxes, the employment opportunities within the tobacco industry, the weak anti-tobacco legislations and public health campaigns against aggressive marketing, relatively low price and pervasive accessibility of tobacco products are considered to drive the tobacco epidemic in Indonesia ^4– 6^. Indonesia is also the only country in the Asia Pacific that has not ratified the World Health Organizations (WHO) Framework Convention on Tobacco Control (FCTC) that addresses the demand and supply of tobacco and related products in order to promote health ^3^.

This weak implementation of international regulations to minimize the marketing, distribution and sales of tobacco products along with prevailing economic, political and social factors have resulted in a high burden of tobacco-related morbidity and mortality in Indonesia ^4^. As a consequence, >97 million non-smokers are exposed to second-hand tobacco smoke in the country ^7^. In 2010, an estimated 12% of total deaths in Indonesia were the result of tobacco-related disease ^8^. Greater than 3.5 million disability-adjusted life years (DALY) were also lost and an estimated 319 million to 1.2 billion USD was spent on healthcare for tobacco-related illnesses in Indonesia annually ^7, 8^. Despite this large burden the support available to quit smoking, especially through the healthcare system, is limited ^8^. This is evidenced by the fact that a third of the patients with TB attempting to quit relapsed into smoking six months after treatment ^9^.

In Indonesia, nicotine replacement therapy such as nicotine patches, gums, and sprays are available without prescription in pharmacies but cost 7000–25000 IDR (0.0035–0.035 USD) per unit. Quitting with prescription medication such as varenicline, hypnotherapy or behavioral therapy costs 0.8–1 million IDR and are not very popular ^8^. Further, most (71%) of those who want to quit smoking, attempt to quit without assistance while others may use traditional methods (herbal or medicinal plants), smokeless tobacco or counselling ^8^. The costs of quitting against the costs of cigarettes (12–24000 IDR/pack currently and 32–53000 IDR from 1 ^st^ November 2019) probably influences both the quit strategy and the decision to continue smoking. The self-confidence of Indonesian physicians in providing smoking cessation counselling is reportedly low, with smoking cessation services offered only at a few healthcare facilities ^10^ while no national toll-free quit-line is available ^11^.

Further, there are varied perceptions of the effects and complications of tobacco on health in Indonesia. For example; some patients with diabetes mellitus considered that they could smoke relatively lesser cigarettes (3/day) when compared with those who were healthy (12/day) ^12^ while others it did not know that smoking could complicate their illness ^13^. Also, quitting smoking was considered an option only for those seriously ill which could be resumed on recovery ^12^. This prevailing scenario makes innovation and improvement in smoking cessation interventions in Indonesia a necessity.

The increasing use of mobile phones in resource poor settings and their adoption for healthcare delivery popularly known as mHealth ^14^, provides an ideal opportunity to deliver smoking cessation interventions in any setting. mHealth supports a wide range of healthcare applications ^15^ including clinical decision support and healthcare data collection ^16^. Other mobile phone applications include behavior change interventions for medication adherence support and smoking cessation ^16, 17^.

Using mobile phones in smoking cessation programs enables the personalization of smoking cessation support based on the quitter’s background, time of the day or the location of the quitter. Short Messaging Service (SMS) Multimedia Messaging Service (MMS) (pictorial or messages or videos), live-voice calls and interactive voice response (IVR) technology that replace a human caller with a computer, provide motivation and counseling to those who want to quit smoking ^15^. Such messages might use prompts (either text i.e.; SMS, picture i.e, MMS or voice i.e., calls and IVR) to encourage avoiding cigarettes, ashtrays, lighters, and environments where people usually smoke eg.; ‘For the next 4 hours, stay away from cigs’. Additionally, messages also help identify the challenges to quitting and plans to overcome them. Prompts (either SMS or voice calls/IVR) to use telephone helplines and nicotine replacement therapy ^18^, information regarding economic savings from quitting and nutrition are also useful.

In this regard, text messaging was effective for smoking cessation in New Zealand (personalized text messages to provide distraction, advise and support) and the United Kingdom (motivational messages and feedback focusing on their achievements), mobile phone applications, though not tested for efficacy in a randomized control trial (RCT), are known to reach smokers who are not seeking professional help ^18– 23^.

A study from United Kingdom (UK) showed that motivational messages encouraged those wanting to quit smoking by focusing on their achievements. They also provided positive feedback, emphasized on the benefits of quitting, consequences of smoking and the process of quitting ^24^.

Personalized text messages were used to provide smoking cessation advice, support, and distraction from smoking in a study from New Zealand. Content covered symptoms expected on quitting, tips to avoid weight gain and improve nutrition, tips to cope with craving; advice to avoid smoking triggers; instructions on breathing exercises to perform instead of smoking and motivational support and distraction ^22^.

### Mobile phone penetration and mHealth development in Indonesia

The growth of mobile users in Indonesia is one of the fastest in Asia with a steady increase from 125 per 100 people in 2013 to 173 per 100 people in 2018 ^25^. Given the improving internet accessibility and low cost of smartphones ^26^, with prices as low as 40 USD for a phone, smartphone penetration in Indonesia has reached 27% in 2018 ^27^ and is predicted to reach 32% by 2022 ^28^. The abundant use of mobile phones in Indonesia that parallels the tobacco epidemic in the country makes mobile phones deemed ideal for implementing smoking cessation interventions.

While mHealth is rapidly evolving in high income countries, scientific evaluation of mobile phone use for health care, particularly for smoking cessation interventions in Indonesia is still in inception ^29, 30^. However, it is essential to first explore the acceptability and perceived usefulness of receiving a mobile based smoking cessation intervention among Indonesians who smoke prior to developing and testing such an intervention. We therefore, chose to determine the preferred mode of communication, potential content and communication characteristics of mobile phone-based smoking cessation interventions prior to developing such an intervention. To our knowledge this is the first study that has assessed the acceptability of mobile phone applications for smoking cessation in Indonesia.

## Methods

This was an exploratory cross-sectional web-based survey conducted in Indonesia between March 23 ^rd^ to April 21 ^st^ 2015. As we did not have prior data on acceptability of mobile phone interventions in Indonesia, we did not estimate a sample size for the study.

For the survey, we developed a survey questionnaire and made it available via the internet for respondents to fill (Appendix A and B (Extended data ^31^). The questionnaire was ‘face validated’ for content and comprehension and was made available in the Indonesian language. The snowballing approach was used to distribute the questionnaire. For this, 25 potential respondents known to the first author were invited to participate in the study. On completing the survey questionnaire these respondents were requested to invite contacts who in turn were requested to invite their contacts and so forth. The respondents could access the questionnaire only on expressing consent to participate in the survey by clicking the AGREE button on the survey web page.

The survey was promoted via a weblog called BerhentiMerokok.org meaning “quit smoking”. This website was created to provide respondents information about the study, to enable respondents to refer the questionnaires to other potential participants and to communicate with the researcher. Another website and a Facebook page “Layanan Online Berhenti Merokok” (“Quit Smoking Online Information”), also promoted the survey (Appendix C (Extended data ^31^). The websites and Facebook page were further promoted via internet based smoking cessation campaigns and health promotion programs using websites, social media accounts and mobile applications using promotional banners (Appendix D (Extended data ^31^)).

The questionnaire comprised four sections: (i) Introduction and informed consent (ii) demographic characteristics (iii) smoking status and smoking cessation aid seeking behavior (iv) mobile phone usage, perceived usefulness and preferences regarding mobile phone-based smoking cessation interventions. The survey included questions on the preferred mode of communication for the intervention delivery (SMS, voice calls, multimedia messaging, automated calls and smartphone applications), potential content and communication characteristics of a smoking cessation intervention delivered via mobile phones. The questionnaire was created using Typeform survey software and was made available online for data collection during the study period (Appendix E (Extended data ^31^)).

During the four-weeks of data collection (March 23 ^rd^ to April 21 ^st^ 2015), 850 visitors had accessed the web-based survey. Of these, only 161 (19%) completed the survey and were included in the analysis. These respondents were current and former smokers, aged 18 years or older, residing in Indonesia for the past year. On average the participants’ took 34:06 (±2:02) minutes to complete the questionnaire.

### Statistical analysis

Statistical analyses were performed using SPSS Version 22 for Windows. Complete case analysis was used to analyse the data. The variables were described using measures of central tendency and dispersion. Bivariate analysis (chi-square) was used to explore associations between perceived usefulness of receiving intervention and demographic variables, smoking status and mobile phone usage. Univariate logistic regression analyses were performed if the variable had more than three categories. Variables with p-values less than 0.25 were subsequently included in a multivariate regression model to identify the predictors of perceived usefulness of the intervention.

### Ethics statement

Ethical clearance for the study (Ref: KE/FK/311/EC) was obtained from the Medical and Health Research Ethics Committee, Universitas Gadjah Mada, Yogyakarta, Indonesia, a state-owned university to which the researchers are affiliated (Appendix F (Extended data ^31^)). Informed consent was obtained online prior to the survey by asking those willing to participate in the survey to click on an “AGREE” button online.

## Results

Of the, 161 (19%) respondents who completed the survey, 47 (29%) used smartphones, 30 (19%) personal computers, and 24 (15%) used tablets. Respondents’ locations represented 14 of the 34 provinces in Indonesia (see Underlying data ^32^).

## Perceived usefulness of receiving a smoking cessation intervention via mobile phones

Overall, 116 (85%) of the respondents perceived that a potential smoking cessation intervention delivered via mobile phones was useful.

***Socio-demographic characteristics*.** The socio-demography of the respondents is described in Table 1. The mean age of the respondents was 29.4 (±7.11) years. Of the 123 respondents, 80 (65%) were aged < 30 years, 102 (83%) were men, 75 (61%) reported Indonesian as their primary language and 68/156 (44%) reported being literate in English. Most respondents were unmarried and had completed higher education. There were 96 (88%) respondents from urban areas. The respondents’ mean monthly expenditure was 4.7 million Indonesian Rupiah (IDR) (± 6.4 million) [USD 330 (± 450)]. There was no significant difference in the perceived usefulness of receiving smoking cessation intervention via mobile phones within different socio-demographic groups ( Table 1 & Table 2).

**Table 1.** Demographic profile of the participants.

Variables		Total	Female (n=22)	Male (n=102)	*P* value
**Age (n=123)**	Median (IQR)	27 (25-32) years	26.5 (23.75- 30.25) years	27 (25-33.5) years	
	≥27 years	70 (57%)	11 (50%)	59 (58%)	
	<27 years	53 (43%)	11 (50%)	42 (41%)	0.470
**Marital status** **(n=121)**	Married	50 (41%)	6 (27%)	44 (43%)	
	Single	71 (59%)	16 (73%)	55 (54%)	0.139
**Residence (n=123)**	Rural	27 (22%)	4 (18%)	23 (23%)	
	Urban	96 (88%)	18 (82%)	78 (76%)	0.637
**Education status** **(n=123)**	High school and lower	17 (14%)	4 (18%)	13 (13%)	
	Undergraduate degree	79 (64%)	16 (73%)	63 (62%)	
	Postgraduate degree	27 (22%)	2 (9%)	25 (25%)	0.142
**English Literacy** **(n=156)**	No	88 (56%)	6 (27%)	48 (47%)	
	Yes	68 (44%)	16 (73%)	52 (51%)	0.076
**Employment status** **(n=123)**	Not gainfully employed	37 (30%)	6 (27%)	31 (30%)	
	Gainfully employed	86 (70%)	16 (73%)	70 (69%)	0.751
**Income (in IDR)** **(n=120)**	Median (IQR)	3000000 (2000000- 5000000)	5000000 (2000000- 7125000)	3000000 (1925000- 5000000)	
	≥3000000 IDR	65 (54%)	15 (68%)	50 (49%)	
	<3000000 IDR	55 (46%)	7 (32%)	48 (47%)	0.144

IDR: Indonesian Rupiah. 1 USD = 14270 IDR as of August 5th, 2019.

**Table 2.** Demographic profile and its association with perceived usefulness of receiving smoking cessation intervention via mobile phone (N=122).

Variables		Total n (%)	Perceived as useful n (%)	P- value	Unadjusted OR (95% CI)
**Age** **Median ± SD (years)**		27 ± 7.11		0.887	0.995 (0.927- 1.068)
**Sex**	Male	101 (82)	86 (85)		Referent
	Female	22 (18)	20 (91)	0.478	1.744 (0.369- 8.247)
**Marital Status**	Married	49 (41)	41(84)		Referent
	Single	71 (59)	62 (87)	0.573	1.344 (0.479- 3.768)
**Primary** **Language**	Indonesian	75 (61)	65 (87)		Referent
	Regional language	47 (39)	40 (85)	0.809	0.879 (0.310- 2.495)
**Education** **Status**	High School and Lower	17 (14)	16 (94)		Referent
	Undergraduate Degree	79 (64)	65 (82)	0.279	0.313 (0.038- 2.568)
	Postgraduate Degree	27 (22)	24 (89)	0.563	0.500 (0.048- 5.242)
**English Literacy**	Illiterate	66 (50)	55 (85)		Referent
	Literate	67 (50)	58 (87)	0.602	1.289 (0.496- 3.350)
**Employment** **Status**	Unemployed	36 (30)	33(92)		Referent
	Employed	86 (70)	72(84)	0.248	0.468 (0.126- 1.738)
**Monthly Expense** **Median ± SD (in IDR)**		3,000,000 ± 6,358,018		0.253	1.000 (1.000- 1.000)
**Residence**	Municipality	95 (78)	81 (85)		Referent
	Regency	27 (22)	24 (89)	0.631	1.383 (0.367- 5.215)

IDR: Indonesian Rupiah. 1 USD = 14270 IDR as of August 5th, 2019.

***Smoking status characteristics*.** Of the respondents, 111 (75%) were current smokers. Of these, 77 (52%) smoked daily. The mean age at which smoking was initiated was 16.55 (± 5.2) years. The mean duration of smoking was 8.5 (± 7) years while most were at a low or very low nicotine dependency as per the FTND. Participants reported smoking an average 9 (SD8) tobacco sticks/ day (range <1–32 tobacco sticks/ day) amounting to 3 pack years (range: 0.1–41 pack years).

Most current smokers (76, 68%) expressed their willingness to quit smoking and a majority (82, 74%) tried to quit in the past. Willingness to quit smoking was an important factor for perceived usefulness of an intervention. Details regarding smoking cessation methods used, the frequency of the health care provider enquiring about the smoking status, and the frequency of advice received to quit are described in Table 3.

**Table 3.** Smoking status characteristics and its association with perceived usefulness of receiving smoking cessation intervention via mobile phone (N=136).

Characteristics		Frequency n (%)	Perceived usefulness n (%)	P- value	Unadjusted OR (95% CI)
**Age at first smoke: Mean ± SD** **(years)**		16.55 ± 5.2		0.989	1.001 (0.913- 1.097)
**Length of active smoking: Median** **± SD (years)**		7 ± 7.0		0.835	0.993 (0.932- 1.059)
**Smoking status**	Daily smoker	77 (52)	61 (79)		Referent
	Occasional smoker	34 (23)	26 (76)	0.948	0.959 (0.271- 3.395)
	Former smoker	38 (26)	29 (76)	0.243	0.535 (0.187- 1.528)
**Duration of abstinence: Median ±** **SD (years)**		3 ± 6.9		0.605	0.978 (0.900- 1.063)
**Nicotine Dependence Level** **(Fagerstrom)**	Very Low and Low	38 (59)	33 (89)		Referent
	Medium, High and Very high	27 (41)	23 (85)	0.632	0.697 (0.158- 3.076)
**Smoking cessation attempt within** **last 12 months**	Yes	76 (76)	69 (91)		Referent
	No	24 (24)	18 (75)	0.045	0.304 (0.091- 1.018)
**Smoking cessation method used in** **the past 12 months**	Quitting with assistance	7 (9)	5 (71)		Referent
	Quitting without assistance	69 (91)	58 (84)	0.569	1.933 (0.193- 19.394)
**Source of quit smoking advice**	Motivation from others	35 (35)	30 (86)		Referent
	Self-motivation	65 (65)	57 (88)	0.779	1.118 (0.357- 3.949)
**Frequency being asked regarding** **smoking status by health provider**	Every time	19 (17)	13 (68)		Referent
	Sometimes	45 (41)	38 (84)	0.397	2.019 (0.397- 10.272)
	Never	45 (41)	35 (78)	0.625	1.462 (0.319- 6.698)
**Frequency being advised to quit** **smoking by health provider**	Every time	14 (13)	9 (64)		Referent
	Sometimes	38 (35)	33 (87)	0.676	1.467 (0.243- 8.854)
	Never	57 (52)	44 (77)	0.585	1.630 (0.282- 9.412)
**Willingness to Quit Smoking**	No	13 (15)	8 (62)		Referent
	Yes	76 (85)	69 (91)	0.004	6.161 (1.579- 24.033)

Of those who tried quitting smoking, 69 (91%) attempted quitting without assistance. Respondents who were willing to quit smoking were seven times more likely to perceive receiving a smoking cessation intervention via mobile phones as useful (OR=6.161, p-value=0.004) ( Table 3).

***Mobile phone usage patterns*.** Of the respondents, 154 (98%) used a smartphone and none of them shared their phones with others. Nearly all phone use was meant for personal reasons (153, 95%). Three-quarters (118, 77%) of the respondents reported being well acquainted with using mobile phones. Most respondents (140, 92%) had uninterrupted internet access via data services on their mobile phones.

Of the respondents, 95 (77%) used their mobile phone to communicate with others for health purposes. These respondents frequently communicated with physicians (33, 35%), health care workers (13, 14%), family (60, 63%) and friends (56, 59%) for health. The content of these communications included request for advice regarding management of illness (51, 32%) and medication side effects (29, 18%), reporting symptoms (46, 29%), scheduling appointments (9, 6%), advising other regarding healthcare (27, 17%), and exchanging information regarding smoking cessation support (22, 14%).

Of the respondents, 85 (89%) who had used a mobile phone for health purposes perceived a potential smoking cessation intervention via mobile phones as useful (OR =3.598, p-value=0.014) ( Table 4).

**Table 4.** Mobile phone ownership and usage patterns and its association with perceived usefulness of receiving a potential smoking cessation intervention via mobile phone (N=137).

Characteristics		Frequency n (%)	Perceived usefulness n (%)	P- value	Unadjusted OR (95% CI)
Mobile phone use proficiency*	Average	35 (23)	25 (71)		Referent
	Good	68 (44)	57 (84)	0.441	1.629 (0.471-5.629)
	Excellent	50 (33)	34 (68)	0.650	0.756 (0.226-2.531)
**Monthly expense for mobile** **phone: Median ± SD (IDR)**		150,000 ± 248,413		0.741	1.000 (1.000-1.000)
**Main use of mobile internet**	Browsing	62 (41)	46 (74)		Referent
	Text-messaging	49 (33)	35 (71)	0.459	0.685 (0.251-1.865)
	Accessing email and other applications	39 (24)	34 (87)	0.140	3.326 (0.675-16.392)
**Alarm use**	No	18 (12)	15 (83)		Referent
	Yes	132 (88)	101 (77)	0.283	0.337 (0.042-2.696)
**Prior mobile phone use for** **health communication**	No	29 (23)	21 (72)		Referent
	Yes	95 (77)	85 (89)	0.014	3.598 (1.240-10.441)

IDR: Indonesian Rupiah. 1 USD = 14270 IDR as of August 8th, 2019.

A multivariate logistic regression analysis of perceived usefulness of mobile phone smoking cessation interventions found only willingness to quit smoking as a predictor of perceived usefulness ( Table 5).

**Table 5.** Multivariate analysis of predictors of perceived usefulness (N =76).

Characteristics	Perceived usefulness (n)	Unadjusted OR 95% CI	Adjusted OR 95% CI
**Employment status**	No (33)		Referent
	Yes (72)	0.468 (0.126-1.738)	0.502 (0.080-3.173)
**Monthly Expense Mean (std)**		1.000 (1.000-1.000)	1.000 (1.000-1.000)
**Smoking cessation attempt** **within last 12 months**	Yes (69)		Referent
	No (18)	0.304 (0.091-1.018)	0.377 (0.087-1.641)
**Willingness to quit smoking**	No (8)		Referent
	Yes (69)	6.161 (1.579-24.033)	5.105 (1.051-24.808)
**Prior mobile phone use for** **health-related communication**	No (21)		Referent
	Yes (85)	3.598 (1.240-10.441)	1.799 (0.386-8.391)

### Features of smoking cessation interventions via mobile phones preferred by respondents

**Figure 1.**
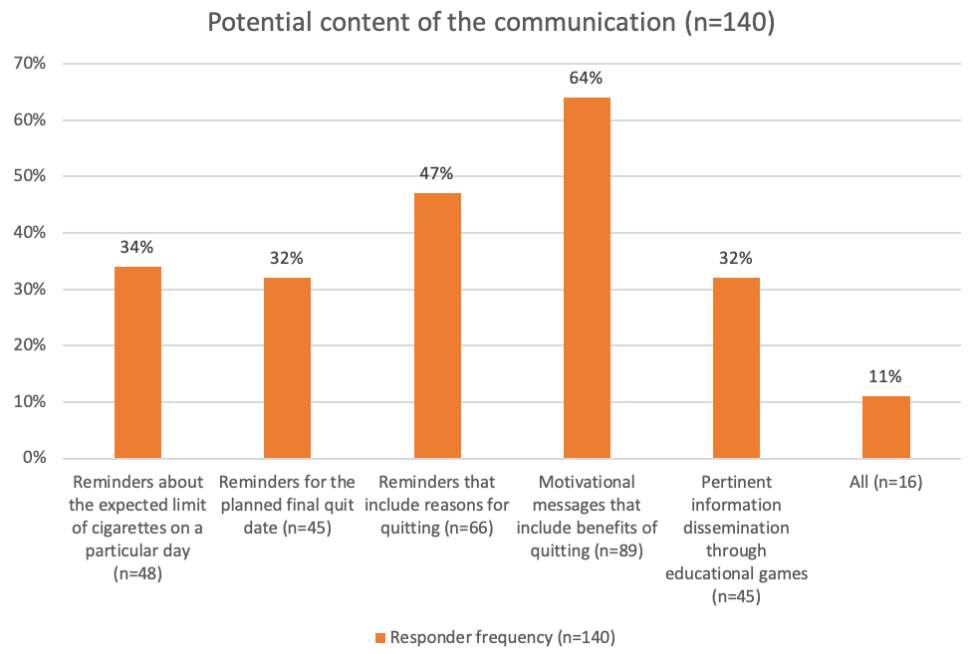
Potential content of the communication (n=140)

***Content and mode of communication*.** Of the respondents, 86 (62%) preferred a smartphone application as a potential smoking cessation intervention as opposed to 18% who were willing to have SMS or MMS for communication ( Figure 1).

**Figure 2.**
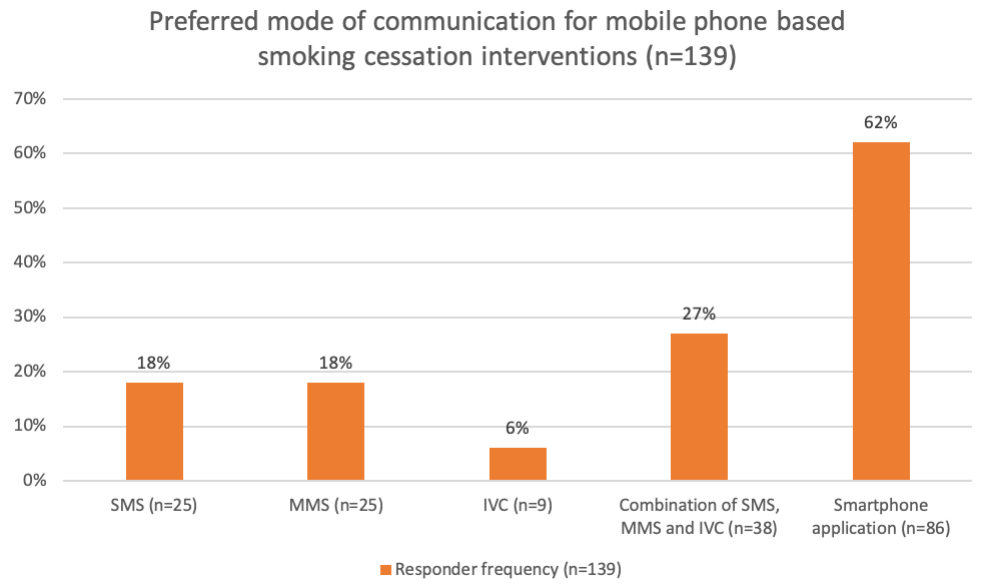
Preferred mode of communication for mobile phone based smoking cessation interventions (n=139). SMS: Short Message Service; MMS: Multimedia Messaging Service; IVR: Interactive Voice Response.

As for the content, motivational messages were the preferred content for mobile phone based smoking cessation interventions, followed by reasons for quitting and reminders about the the numbers of cigarettes that they could smoke each day as they approached their quit date ( Figure 2).

***Characteristics and features of communication*.** Two communication characteristics relevant for smoking cessation were explored, i.e., interactivity and personalization. Nearly half the respondents (65, 47%) preferred partially interactive communication, 41 (30%) preferred completely interactive communication and the rest (32, 23%) requested a non-interactive one-way communication. Personalization of content to their needs was a necessary feature for 126 (91%) respondents, while 84 (61%) requested interventions delivered at customized times.

Most respondents (55, 40%) wanted to receive smoking cessation communication on demand and throughout the day (37, 46%).

**Figure 3.**
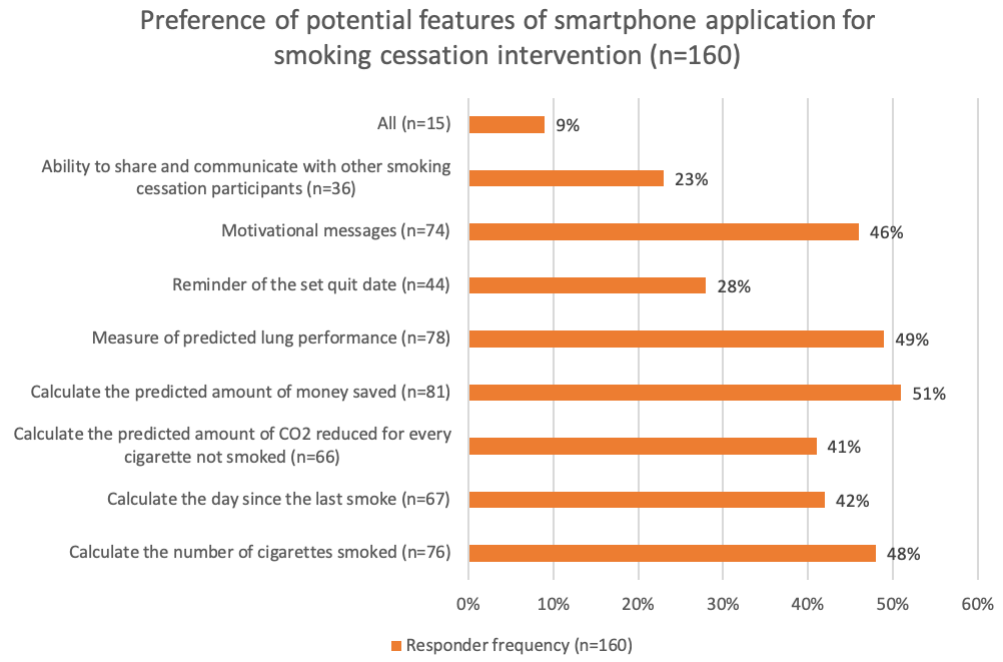
Preference of potential features of smartphone application for smoking cessation intervention (n=160).

Potential features of the smartphone application for smoking cessation application requested are described in Figure 3. A calculator indicating the amount of money saved was the most popular followed by predicted lung performance and motivational messages.

## Discussion

In Indonesia, an LMIC, the burden of tobacco smoking has risen from 59 million in 2000 to 70 million in 2010 and 73.6 million in 2015 ^33, 34^ and continues to rise, increasing the financial burden due to tobacco related illnesses. The situation is compounded by the limited awareness regarding the hazards of smoking along with the minimalistic support available to quit. Currently, 10% of tobacco users quit tobacco annually ^35^. Further, literature from Indonesia shows that 66% of research participants in one study unsuccessfully attempted to quit smoking ^36^, while another study reported that 15% its participants had quit smoking ^37^. To address this issue innovative solutions that are acceptable to Indonesians wanting to quit is essential. Therefore, given the current pervasiveness of mobile phone communication and its affordability we sought to explore the acceptability and design for a mobile phone smoking cessation intervention in Indonesia.

### Quitting with mobile phones

No universally effective intervention to address the tobacco epidemic exists. While willingness to quit smoking is a necessity, life-altering events also known as ‘teachable moments’ also lead to quitting ^38, 39^. Behavior change interventions such as the counselling, self-help materials, physicians brief advice, telephone calls and pharmacotherapy are interventions commonly used in quitting ^40^. In addition, the rapid uptake of information technology (IT) has spurred innovative ways to support quitting via mobile phones.

Currently in Indonesia behavior change interventions are uncommon and when available are expensive. So also, is advice from physicians regarding quitting. Our study indicated that nearly half the participants did not receive any advice from their healthcare provider to quit, despite a reported desire to do so.

**Figure 4.**
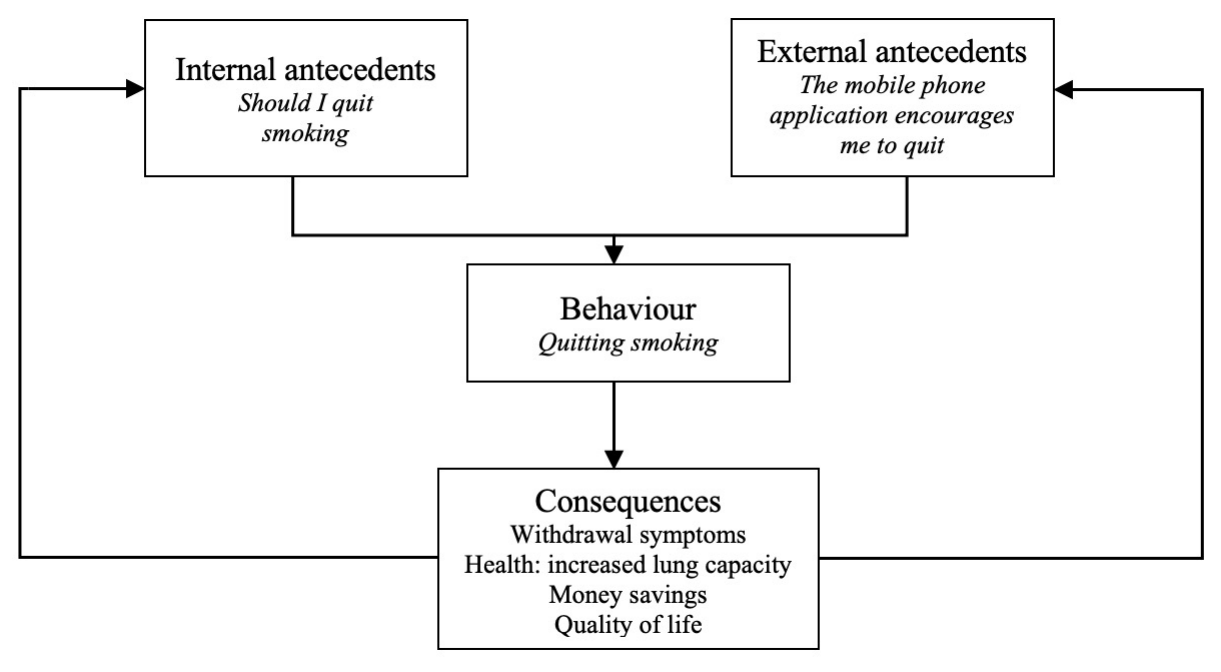
Behavior learning theory.

In this scenario, integrating mobile phones into the behavior learning theory (BLT) ^41^ provides a theoretical model for mHealth interventions in smoking cessation. Based on BLT, quitting results from combined external antecedents or motivators (mHealth intervention) and internal antecedents (willingness to quit). Positive outcomes i.e., better health, money savings and better quality of life sustain quitting by reinforcing willingness and engagement with the intervention ( Figure 4).

### Perceived usefulness of a potential smoking cessation intervention via mobile phones

Studies globally have found smoking cessation intervention via mobile phones ^42– 45^ are feasible and acceptable to young people across different socio-economic groups. In our study, such interventions were more likely to be perceived as useful by respondents willing to quit smoking. An earlier study found smartphone applications were more frequently used by respondents who were willing to quit within 30 days ^19^.

Though we did not find additional evidence, our study showed that respondents who had used a mobile phone for health-related communication perceived a smoking cessation intervention via mobile phones as useful. This was probably due to their experience and comfort with interventions delivered via mobile phones.

### Features of smoking cessation interventions via mobile phones preferred by respondents

***Mode of communication*.** Our study suggests that a smartphone application is the most preferred mode of communication for a potential smoking cessation intervention. A few respondents chose SMS, MMS, IVR or a combination of the three as the mode of communication. The larger percentage of respondents having access to the internet may explain this result. Given the improving internet accessibility and smartphone subscription in Indonesia ^25^, smartphone applications might be the most suitable mode of intervention for smoking cessation.

Further, literature showed that some of the widely used modes for delivery of health interventions via mobile phones were SMS and tele-counselling while MMS was not as widely used and tested as the other modes of communication ^16^. SMS interventions are found effective in various behavioral change interventions such as diabetes self-management, weight loss management, physical activity, smoking cessation and medication adherence for antiretroviral therapy ^46^. SMS or text-message-based smoking cessation intervention is the only mobile phone-based intervention that was effective in randomized trials. Studies in the United Kingdom and New Zealand reported that text-message-based smoking cessation interventions are affordable, can be personalized, are age appropriate, and not location dependent ^18, 22^. In Indonesia, no costs are incurred to receive a text message, while it costs IDR 360 IDR (3 cents) to send an individual message and when deployed in bulk, messaging would cost up to IDR 65,000 (USD 4.5) for 500 messages a month to be incurred by the intervention provider.

A study in New Zealand showed that an MMS-based smoking cessation intervention using video messages was effective. The results however were equivocal when a complex video messaging intervention was compared with simple general health videos that communicated general health messages. Video messaging was not considered economical in all socioeconomic groups, even in resource rich settings such as New Zealand ^44^. Another randomized trial that used multiple-component personalized counselling via telephone in high school students in the US showed an increase in abstinence rates ^47^. To deal with the problem of tobacco epidemics, many high-income countries have also established several tele-counselling interventions such as the “Quit Line” and incorporated it with the national health service. Such a quitline is not available in Indonesia, despite the large tobacco epidemic.

Smartphone applications are a promising medium to reach smokers across multiple nations. They have the potential to consolidate the advantages of smoking cessations interventions designed for use with or without the internet (i.e., computer based). Users can continue to access motivational features such as calculators for money saved per cigarette not smoked or information downloaded and saved within the applications from the internet. Mobile applications can be designed successfully to harness mobile phone features such as video, audio, interactive media and texting to promote engagement and constant motivation to quit smoking to the users ^19^. Given the preference for smartphone applications for smoking cessation in our study, an application that uses pre-recorded audio and video based motivational messages could be useful. However, interventions designed should be contextual as the preference for smartphone applications over other forms mobile interventions, such as text messages, might vary globally ^48^.

***Potential content*.** Our study found that motivational messages such as the benefits of quitting smoking and reminders about the users’ reason to quit smoking were preferred content for mobile phone-based smoking interventions in Indonesia. Motivation is the core of any smoking cessation intervention along with addressing barriers and benefits of quitting. The interventions also focus on providing cues to action and promoting self-efficacy and harness the theories of behavior change.

A study from the United Kingdom (UK) showed that motivational messages encouraged those wanting to quit smoking by focusing on their achievements ^18, 24^. Personalized text messages were used to provide smoking cessation advice, support, and distraction from smoking in a study from New Zealand. Content had also covered symptoms expected on quitting, tips to avoid weight gain and improve nutrition, coping with craving; advice to avoid smoking triggers; instructions on breathing exercises to perform instead of smoking and motivational support and distraction ^22^.

***Communication characteristics*.** We explored two important characteristics namely, interactive communication and personalized communication. Most respondents preferred to interact with a human facilitator and wanted personalized communications. They preferred receiving messages on demand or even throughout the day without a predetermined frequency. Though other studies have not discussed the timing of communication delivery, most interventions involved predetermined daily communication.

Although communication in the UK and New Zealand studies was an automated SMS, both interventions allowed participants to contact a quit line and speak to a counsellor at any time. Additionally, the intervention in New Zealand allowed the respondents to send free messages to friends and family in order to obtain support ^22^. Earlier studies about the social network structure of large online communities for smoking cessation have shown a relationship between social network support for quitting and maintenance of abstinence ^49^. Higher levels of connectivity and positive social support are known associates of a greater quit rate and lower rate of relapse ^50^. Therefore, mobile-phone quit interventions should incorporate an interactive component to enable quitting in their design.

Both the UK and New Zealand interventions combined interactive and one-way communication. Although most text messages sent to the participants were push messages, the UK- study provided a “CRAVE” and “LAPSE” feature, where the participants could ask for additional messages ^18^. Similarly, the New Zealand-based study provided a “txt crave” feature where participants could ask for additional messages during their moments of craving and the “txt quiz” feature where the participants could ask questions ^22^.

Several studies have explored the effectiveness of personalised interventions for smoking cessation ^51– 55^. Improved engagement and retention through mobile-based smoking cessation interventions in adolescents has been observed ^42, 55, 56^. Some studies used personalized messages ^18, 42, 44, 54, 57^. Participants sex, age, smoking history, goals, medical condition ^16, 42, 52^ cultural and ethnic background ^53, 58^ are some factors used in personalising messages. The profound ethnic diversity of the Indonesian population, if considered, might increase the complexity of the intervention and costs for development ^59^.

### Features currently available in mobile applications for smoking cessation

Smoking cessation applications are pervasive, some with exaggerated claims of effectiveness. Despite the large number of smartphone applications for smoking cessation ^20, 60^, only a few are evidence-based ^20^ and are insufficient to stimulate self-motivation ^60^ to help quit smoking.

In 2012, an American-based survey analyzed 98 of the most popular smartphone applications for smoking cessation (available in English) downloaded via the iPhone and Android market. Popular applications had low levels of adherence to the U.S. Guidelines for Treating Tobacco Use and Dependence (GTTUD), with an average score of 12.9 of a possible 42 on the Adherence Index ^20^.

While the applications incorporated features such as instructiveness, user personalized advice to quit and assessment of current tobacco use, motivation through rewards, and quit plan assistance were missing. Additionally, advise for referral and follow-up were also missing ^61^.

The list of potential smartphone features, in various combinations, for mobile phone interventions is exhaustive ^19, 20, 60^. One such feature is the interactive self-monitoring system that allows users to add their health data via questionnaires, texts, and audio or video recordings ^20^. These applications process, organize and graph this data to help users understand their progress. The data can help the users at every step in their quitting process, providing text information about quitting, showing the number of days users have been nicotine-free, providing logs to administer users’ quit attempts and craving triggers along with sending them motivational messages and reminders ^19^.

Some of the least explored features of mobile smoking cessation applications such as a calculator for predicting money saved from quitting and unsmoked cigarettes along with predicted lung function were features popular in our study. However, this may also be due to the structure of the questionnaire and the nuance of smartphone-based quitting applications in Indonesia.

### A conceptual framework for designing mobile phone smoking cessation interventions

**Figure 5.**
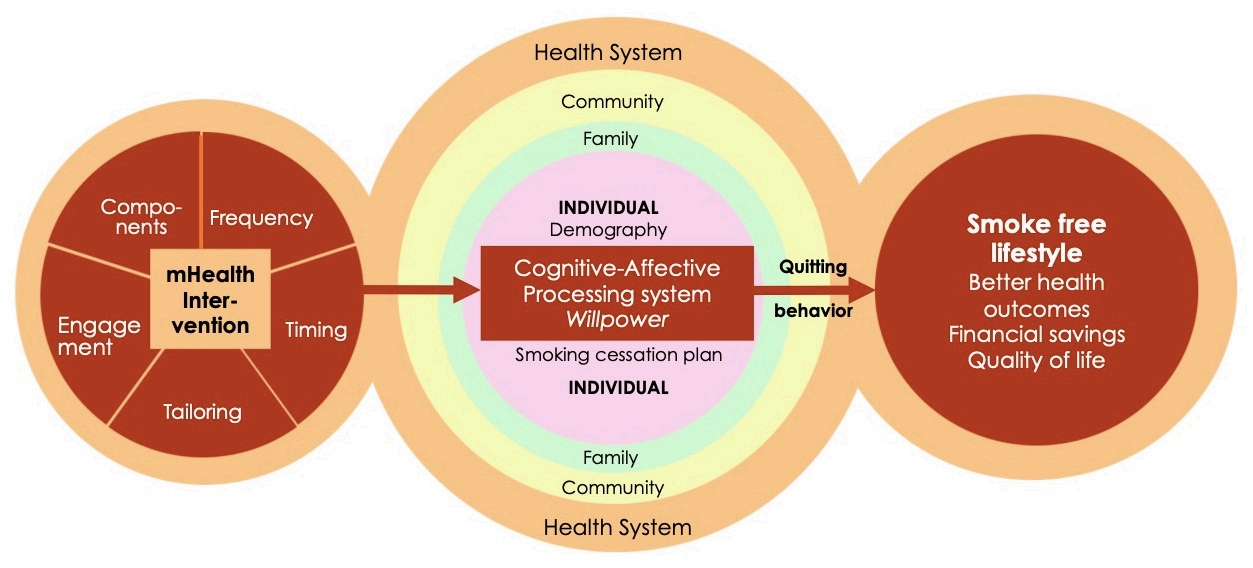
Conceptual model for mHealth interventions in smoking cessation. This figure has been reproduced with permission from Rodrigues R (2014) ^62^.

Based on the results we modified the conceptual framework for mHealth interventions by Rodrigues R (2014) ^62^ to inform mHealth intervention design for smoking cessation ( Figure 5). Such interventions should consider frequency, timing, personalization (tailoring) engagement and components (features, single or multiple) in their design. For example; an mHealth smoking cessation intervention could provide timed motivational messages, distractions from craving, reinforcements such as graphic visualizations of money saved based on interactive data input from users. Further, the Cognitive-Affective Personality System (CAPS) model provides a possible mechanism to incorporate the intervention for behavior change ^63, 64^. CAPS is a complex network of an individual’s goals, beliefs, thoughts, feelings, self-regulatory standards, plans and competencies. An individual’s thoughts and feelings are constantly changing. External stimuli through mobile phones (messages and prompts) can trigger these changes thereby influencing self-regulatory behavior.

### Limitations

As this was a web-based study only those who were familiar with the internet were captured minimizing its generalizability to those familiar with information technology (IT). Nevertheless, as the proposed intervention is IT based, it captured the opinion of the beneficiaries that the intervention is likely to target. Also, as not all who accessed the questionnaire completed it, the numbers that were included in the analysis were low. However, despite the study’s limited sample size and duration, information relevant to inform the design and piloting the mobile application was obtained. Item non-response and incomplete responses are known to affect the generalizability of the results of online surveys ^65, 66^. As the reported levels of tobacco dependency were low, it is likely that the respondents were those who either had greater control over their smoking behavior or were more amenable to the idea of quitting. However, the level of nicotine dependence obtained may be questionable given the normalization of smoking in Indonesia ^5, 67^. Further, social desirability bias also cannot be ruled out in the FTND as it is a self-report of dependence by the participant.

Given that quitting cold turkey is a popular method of smoking cessation we understand that our approach to smoking cessation reflects a dominance of interventionism. The proposed intervention should not be considered as a ‘one size fits all’ but rather one in a basket of solutions including behavioral therapy, pharmacotherapy or quitting cold turkey.

## Conclusions

Our study showed that the Indonesian respondents to our survey perceived a potential smoking cessation intervention via mobile phones as useful. Perceived usefulness was associated with smokers’ willingness to quit smoking and their prior use of mobile phones for health-related communication. A multicomponent smartphone application personalized to time, frequency and content was desired. Such an application, if implemented, could be one in a basket of smoking cessation solutions offered within an organized program quit smoking programmes at schools, healthcare facilities and counseling centers could go a long way in addressing the tobacco epidemic in Indonesia.

## Ethics and consent

Ethical approval was received from the Ethics Committee of Universitas Gadjah Mada, Indonesia (Ref: KE/FK/311/EC). Participants were fully informed of the study and consent was obtained prior to data collection.

## Data availability

### Underlying data

Harvard Dataverse: Perceived Usefulness of Receiving a Potential Smoking Cessation Intervention via Mobile Phones among Smokers in Indonesia. https://doi.org/10.7910/DVN/N3QQE1 ^32^

This project contains the following underlying data:

- Main SPSS file.tab (SPSS file with underlying data)
- Raw data on MS Excel with codes and keys.xlsx (underlying data in Excel format)
- Table 2 SPSS outputs.spv (Data underlying Table 2)
- Table 3 SPSS outputs.spv (Data underlying Table 3)
- Table 4 SPSS outputs.spv (Data underlying Table 4)
- Table 5 SPSS outputs.spv (Data underlying Table 5)

### Extended data

Harvard Dataverse: Perceived Usefulness of Receiving a Potential Smoking Cessation Intervention via Mobile Phones among Smokers in Indonesia. https://doi.org/10.7910/DVN/EU6DZS ^31^

This project contains the following extended data:

- Appendix A - Questionnaire in English.pdf (Study questionnaire - English)
- Appendix B - Questionnaire in Indonesian Language - Kuisioner Eksplorasi Penerimaan Penggunaan Ponsel untuk Dukungan Berhenti Merokok.docx (Study questionnaire - Indonesian)
- Appendix C - Promotion of survey on Facebook.png (Survey promotion via Facebook)
- Appendix D - Promotion of survey on Doctor Gratis mobile application, guetau.com and Twitter.png (Survey promotion via Doctor Gratis mobile application, guetau.com and Twitter)
- Appendix E - Survey on Typeform website.png (Image of Survey on Typeform)
- Appendix F - IEC and ethics clearance.pdf (Study consent form and approval document)

Data are available under the terms of the Creative Commons Zero “No rights reserved” data waiver (CC0 1.0 Public domain dedication).
