# Supplementary material for: Perceived usefulness of receiving a potential smoking cessation intervention via mobile phones among smokers in Indonesia
Source: Wellcome Open Res. 2020 Aug 19;4:94. Originally published 2019 Jun 11. [Version 2] doi: 10.12688/wellcomeopenres.15135.2 (PMC7531048; doi:10.12688/wellcomeopenres.15135.2)
Supplement: Peer review history

# Peer review: Reviewer response for version 2

**DOI:** 10.21956/wellcomeopenres.16815.r40022

**Published:** 2020-10-01

**Authors:** Mimi Nichter

**Affiliations:** 1 School of Anthropology, University of Arizona, Tucson, AZ, USA

The authors have addressed the issues that were highlighted in the review.

Is the work clearly and accurately presented and does it cite the current literature?

Partly

If applicable, is the statistical analysis and its interpretation appropriate?

Yes

Are all the source data underlying the results available to ensure full reproducibility?

No source data required

Is the study design appropriate and is the work technically sound?

Partly

Are the conclusions drawn adequately supported by the results?

Partly

Are sufficient details of methods and analysis provided to allow replication by others?

Partly

Reviewer Expertise:

I am a medical anthropologist who has conducted extensive research on the development of tobacco cessation programs in Java, Indonesia.

I confirm that I have read this submission and believe that I have an appropriate level of expertise to confirm that it is of an acceptable scientific standard.

# Peer review: Reviewer response for version 2

**DOI:** 10.21956/wellcomeopenres.16815.r40023

**Published:** 2020-09-21

**Authors:** Judith McCool

**Affiliations:** 1 Department of Epidemiology and Biostatistics, School of Population Health, University of Auckland, Auckland, New Zealand

This revised version of the paper is considerably improved. All issues raised were addressed. This paper will add to the growing literature on mCessation; this one in respect to perceived usefulness of receiving text message support to quit smoking. In the interim since submission, there are likely to be several additional articles that would add value to this work, or to future interventions planned by the authors. Noting here that end-user perceptions of value is important but there is an additional issue of sustainability of any mobile based or digital interventions, this is often outside the influence of the end-user and relies on the funder and implementers to commit to long-term delivery and evaluations.

Is the work clearly and accurately presented and does it cite the current literature?

Yes

If applicable, is the statistical analysis and its interpretation appropriate?

Yes

Are all the source data underlying the results available to ensure full reproducibility?

Partly

Is the study design appropriate and is the work technically sound?

Yes

Are the conclusions drawn adequately supported by the results?

Partly

Are sufficient details of methods and analysis provided to allow replication by others?

Partly

Reviewer Expertise:

Global health, tobacco control, mass media communications, social media, behaviour change, Pacific region.

I confirm that I have read this submission and believe that I have an appropriate level of expertise to confirm that it is of an acceptable scientific standard.

# Peer review: Reviewer response for version 1

**DOI:** 10.21956/wellcomeopenres.16514.r36737

**Published:** 2019-11-18

**Authors:** Judith McCool

**Affiliations:** 1 Department of Epidemiology and Biostatistics, School of Population Health, University of Auckland, Auckland, New Zealand

This paper adds practical, current information on what smokers in Indonesia want in terms of support to quit smoking. Tobacco use in Indonesia is a major global health challenge due to the scale of impact. It is the scale of the issue that lends itself to questioning whether mobile devices may boost the reach and accessibility for effective, evidence based smoking cessation support. In a context where most smokers quit without additional aids, it is important to question what mobile or other smoking cessation support is offered and what costs and benefits this adds over and above what is currently in place. This paper examines the perceived usefulness of mobile smoking cessation (mCessation) support for smokers. It is a credit to the research team that they have paused to ask smokers the question "how do you want your cessation support delivered" rather than going ahead with an costly mCessation pilot. Having said that, it is often stated, that "people do not know, what they don't know", in other words hypothetical interventions may not necessarily provide an accurate gauge of potential use or effectiveness of mobile interventions. However, this work also provides an insight into how mobile phones may used to support smokers, in a low-middle income country. This information is likely to add value to other areas of work where mobile has been proven effective. It is also important to share information about the cultural variations in preferences for, and use of, mobile technologies. There is richer granularity in ITC use within countries than what is described in global mobile agency reports. Hence, this work is timely and will add value to others working in the field of mobile and digital health, as well as tobacco control.

***General comments:***

 Introduction: Page 3. The statement regarding the high prevalence of tobacco use in Indonesia was attributed to the size of the population *and* the lack of public health campaigns addressing cessation. Might it also have something to do with tobacco industry interference, low taxation of tobacco and public awareness messages, smokefree environments, banning advertising and promotions? This statement gives the impression that the current rates are attributable to the size of the population and cessation information, exclusively. 

 Introduction Page 3. The WHO FCTC covers more than prevention of update of tobacco by adolescents and protection from second-hand cigarette smoke. It also covers cessation support and other measures that have evidence of impact. The statement on page X could be amended to reflect the broader intention of the WHO FCTC. The statement at the start of the following paragraph captures the challenges, but again does not make any reference to the weak regulation of the tobacco industry and marketing, distribution and sales of tobacco products. I note though that this paper is focused on the potential to increase cessation, but this is confounded by a supportive smoking environment. 

 Introduction, Page 3, paragraph 6. It was noted that the text messages were found to be effective (to support quit attempts) in New Zealand and the United Kingdom, smartphone android applications.... Is it the case that apps were trialled only on android phones? My interpretation is that they were similarly ineffective whether delivered on iPhone or android phones (Abroms *et al*., 2013 ^1^). 

 Methods. It is possible that having the survey promoted via a weblog that is for smoking cessation may have reduced the potential sample size given the study was about what people think they want for support for quitting, not about if they are trying to quit. This may, in addition to other factors, have reduced the final sample size. 

 Ethics statement, page 4. The ethics statement is included here and again at the end of the paper (on page 12), suggest presenting it once only.

 Results: Smoking status characteristics; it was interesting to note that there were no differences in preference for receiving information via mobile device between socio-demographic groups. I also note that there was a low or very low level of dependency reported, despite the length of time that smoking was reported. Was this expected and is it explained in the discussion?

 It was noted that most smokers in Indonesia quit without behavioural or NRT support. What proportion of those who attempt to quit, succeed. Is there any measure of these changes over time in terms of quitting? What were the methods that were used to support smokers to quit cold turkey? Is there any research on this that can shed light on the types of content that might be salient if delivered via mobile phone?

 The finding that suggests a preference for smartphone apps over text message is unique and is inconsistent with other studies which have found little support for apps. I refer to a co-designed intervention using an app for behaviour change in New Zealand with indigenous and Pacific populations (Ni Mhurchu *et al.,* 2019 ^2^) among others. 

 Page 10. The statement that start "Only a few studies have explored the effectiveness of smartphone applications as a behaviour change intervention" is an important point but may need to be updated. There is an increasing number of publications coming through that question the value of apps in health and behaviour change. 

 Conclusion. Suggest revise the statement that a 'potential smoking cessation intervention...' was perceived as useful. 

 The final statement regarding the implementation of the app within an organised programme (via schools etc) was not a conclusion that came through strongly in the data. It might it be that any intervention, whether it is mobile device delivered or not, needs to complement a comprehensive tobacco control programme, backed by government support. Politics had not been mentioned in any detail until this point and seems out of place or underdeveloped at this stage.

 Table 1. What does 'gainfully employed" mean - I am assuming this mean paid or salaried work?

 Income level in IDR - could there be a proxy figure in US or Euro? It was noted earlier that the DALY for tobacco related illness was reported in USD. 

 Table 4. Column 1 - what does the *refer to on Mobile phone use proficiency*. 

 Figure 4. This figure does not substantively add to the paper. A brief description of the theory of behaviour change, and a reference would suffice.

Is the work clearly and accurately presented and does it cite the current literature?

Yes

If applicable, is the statistical analysis and its interpretation appropriate?

Yes

Are all the source data underlying the results available to ensure full reproducibility?

Partly

Is the study design appropriate and is the work technically sound?

Yes

Are the conclusions drawn adequately supported by the results?

Partly

Are sufficient details of methods and analysis provided to allow replication by others?

Partly

Reviewer Expertise:

Global health, tobacco control, mass media communications, social media, behaviour change, Pacific region.

I confirm that I have read this submission and believe that I have an appropriate level of expertise to confirm that it is of an acceptable scientific standard, however I have significant reservations, as outlined above.

## References

[1] : A content analysis of popular smartphone apps for smoking cessation. *Am J Prev Med*.2013;45(6) : 10.1016/j.amepre.2013.07.008 732-6 10.1016/j.amepre.2013.07.008 24237915PMC3836190

[2] : A co-designed mHealth programme to support healthy lifestyles in Māori and Pasifika peoples in New Zealand (OL@-OR@): a cluster-randomised controlled trial. *The Lancet Digital Health*.2019;1(6) : 10.1016/S2589-7500(19)30130-X e298-e307 10.1016/S2589-7500(19)30130-X 33323252

# Response

**Published:** 2020-08-07

**Authors:** Rashmi Rodrigues

**Affiliations:** St John's National Academy of Health Sciences, India

Dear Dr. McCool,

We very much appreciate and thank you for your valuable time towards reviewing our work. We have addressed your comments to the best of our ability and sincerely hope we have done justice to your suggestions on the report. The changes have been mentioned in a point-wise format as mentioned below, and a second version of the manuscript has been submitted to the journal.

We have added information on the sampling strategy, rephrased the table titles and removed associations for which adjusted odds ratios were unavailable among many other minor corrections, in line with the feedback received.

We hope the revised manuscript meets the standards to be considered for a formally approved status on Wellcome Open Research.

Thanking you again,

Sincerely,

Mochammad Fadjar Wibowo

Submitting Author

(on behalf of all the authors)

**RESPONSE TO REVIEWERS:**

EXTERNAL PEER-REVIEWER #1 (Judith McCool, BA, MPH, PhD)

Thank you once again for your comments. We have taken note of your detailed feedback on various sections in the manuscript and have addressed them to the best of our ability. Please find our point-counterpoint responses to your comments below:

 

- Introduction: Page 3. The statement regarding the high prevalence of tobacco use in Indonesia was attributed to the size of the population *and* the lack of public health campaigns addressing cessation. Might it also have something to do with tobacco industry interference, low taxation of tobacco and public awareness messages, smoke-free environments, banning advertising and promotions? This statement gives the impression that the current rates are attributable to the size of the population and cessation information, exclusively.

Response: We have now revised and reorganized the statement to read as follows (Introduction paragraph 2):

The large revenue from cigarettes despite low taxes, the employment opportunities within the tobacco industry, the weak anti-tobacco legislation and public health campaigns are considered to drive the tobacco epidemic in Indonesia.

 

- Introduction Page 3. The WHO FCTC covers more than prevention of update of tobacco by adolescents and protection from second-hand cigarette smoke. It also covers cessation support and other measures that have evidence of impact. The statement on page X could be amended to reflect the broader intention of the WHO FCTC.

 

Response: This statement has been amended as follows:

Indonesia is also the only country in the Asia Pacific that has not ratified the World Health Organizations (WHO) Framework Convention on Tobacco Control (FCTC) that addresses the demand and supply of tobacco and related products in order to promote health ^3^.

 

- The statement at the start of the following paragraph captures the challenges, but again does not make any reference to the weak regulation of the tobacco industry and marketing, distribution and sales of tobacco products. I note though that this paper is focused on the potential to increase cessation, but this is confounded by a supportive smoking environment. 

 

Response: Thank you very much for highlighting this. We have revised the sentence to read as follows:

This weak implementation of international regulations to minimize the marketing, distribution and sales of tobacco products along with prevailing economic, political and social factors have resulted in a high burden of tobacco-related morbidity and mortality in Indonesia ^4^

- Introduction, Page 3, paragraph 6. It was noted that the text messages were found to be effective (to support quit attempts) in New Zealand and the United Kingdom, smartphone android applications.... Is it the case that apps were trialled only on android phones? My interpretation is that they were similarly ineffective whether delivered on iPhone or android phones (Abroms *et al*., 2013 ^1^).

 

Response: Thank you very much for highlighting this. We have now revised our statement as follows:

In this regard, text messaging was effective for smoking cessation in New Zealand (personalized text messages to provide distraction, advise and support) and the United Kingdom (motivational messages and feedback focusing on their achievements), ***mobile phone applications,*** though not tested for efficacy in a randomized control trial (RCT), are known to reach smokers who are not seeking professional help.

You will notice that we have changed the phrase ***smartphone android applications to mobile phone applications.***

 

- Methods. It is possible that having the survey promoted via a weblog that is for smoking cessation may have reduced the potential sample size given the study was about what people think they want for support for quitting, not about if they are trying to quit. This may, in addition to other factors, have reduced the final sample size. 

Response: Thank you for the suggestion. We did receive 800 odd responses, however as described in the results section. However, you comment has now been incorporated into the limitations of the study.

- Ethics statement, page 4. The ethics statement is included here and again at the end of the paper (on page 12), suggest presenting it once only.

Response: The duplicated ethical statement at the end of the article has been removed per your suggestion.

- Results: Smoking status characteristics; it was interesting to note that there were no differences in preference for receiving information via mobile device between socio-demographic groups. I also note that there was a low or very low level of dependency reported, despite the length of time that smoking was reported. Was this expected and is it explained in the discussion?

 

Response: Thank you once again. We do agree that there was no difference in the preference for receiving information between sociodemographic groups. However, as our study questionnaire did explore this further, we regret, that we are unable to comment on the reason for this at this stage.

We have now discussed the low level of dependency in the limitations of the study as follows:

As the reported levels of tobacco dependency were low, it is likely that the respondents were those who either had greater control over their smoking behavior or were more amenable to the idea of quitting. However, the level of nicotine dependence obtained may be questionable given the normalization of smoking in Indonesia.

 

- It was noted that most smokers in Indonesia quit without behavioural or NRT support. What proportion of those who attempt to quit, succeed. Is there any measure of these changes over time in terms of quitting? What were the methods that were used to support smokers to quit cold turkey? Is there any research on this that can shed light on the types of content that might be salient if delivered via mobile phone?

 

Response: Thanks for the suggestion. We have now incorporated the following in the 1 ^st^ paragraph of our discussion:

Currently, 10% of tobacco users quit tobacco annually. [RR1] Further, literature from Indonesia shows that 66% of research participants in one study unsuccessfully attempted to quit smoking, [RR2] while another study reported that 15% its participants had quit smoking. [RR3] 

Unfortunately, we did not explore the preference to quit smoking in our study as we primarily focused on quitting with mobile phones and providing support for the same over an extended period for those who would prefer this method. We do agree that this method is likely to work only if an individual is motivated enough to use it and that the choice of the method belongs to the individual. Given that quitting cold turkey is a popular method of smoking cessation we understand that there is a dominance of interventionism in our approach and have included this in our limitations as follows:

Given that quitting cold turkey is a popular method of smoking cessation we understand that our approach to smoking cessation reflects a dominance of interventionism. The proposed intervention should not be considered as a ‘one size fits all’ but rather one in a basket of interventions that includes interventions such as behavioral therapy, pharmacotherapy or quitting cold turkey.

 

- The finding that suggests a preference for smartphone apps over text message is unique and is inconsistent with other studies which have found little support for apps. I refer to a co-designed intervention using an app for behaviour change in New Zealand with indigenous and Pacific populations (Ni Mhurchu *et al.,* 2019 ^2^) among others.

 

Response: We agree that the preference to smart phone apps over text messages was found in this study. However, we believe that every population is unique, more so indigenous populations. Our attempt through this study was primarily to assess the acceptability of mobile phone interventions primarily for smoking cessation in Indonesia. The results therefore need to be viewed in this context and are therefore not expected to be generalizable to other populations unless they are similar.

We have incorporated your suggestion in the discussion section under “mode of communication” as follows:

However, interventions designed should be contextual as the preference for smartphone applications over other forms mobile interventions, such as text messages, might vary globally[RR4] .

 

- Page 10. The statement that start "Only a few studies have explored the effectiveness of smartphone applications as a behaviour change intervention" is an important point but may need to be updated. There is an increasing number of publications coming through that question the value of apps in health and behaviour change. 

 

Response: Thank you very much for highlighting this. We have rephrased this as follows, “Smartphone applications are a promising medium to reach smokers across multiple nations.”

 

- Conclusion. Suggest revise the statement that a 'potential smoking cessation intervention...' was perceived as useful. 

 

Response: Thank you for the suggestion. We have revised the statement as follows:

Our study showed that the Indonesian respondents to our survey perceived a potential smoking cessation intervention via mobile phones as useful.

 

- The final statement regarding the implementation of the app within an organised programme (via schools etc) was not a conclusion that came through strongly in the data. It might it be that any intervention, whether it is mobile device delivered or not, needs to complement a comprehensive tobacco control programme, backed by government support. Politics had not been mentioned in any detail until this point and seems out of place or underdeveloped at this stage.

 

Response: Thank you once again. We have revised our conclusion keeping in mind that we studied the acceptability of a mobile phone driven smoking cessation intervention as follows:

Our study showed that the Indonesian respondents to our survey perceived a potential smoking cessation intervention via mobile phones as useful. Perceived usefulness was associated with smokers’ willingness to quit smoking and their prior use of mobile phones for health-related communication. A multicomponent smartphone application personalized to time, frequency and content was desired. Such an application, if implemented, could be one in a basket of smoking cessation solutions offered within an organized program quit smoking programmes at schools, healthcare facilities and counseling centers could go a long way in addressing the tobacco epidemic in Indonesia.

 

- Table 1. What does 'gainfully employed" mean - I am assuming this mean paid or salaried work?

 

Response: Yes, the term refers to an employee receiving steady work and payment from an employer.

 

- Income level in IDR - could there be a proxy figure in US or Euro? It was noted earlier that the DALY for tobacco related illness was reported in USD. 

 

Response: A conversion for IDR and USD is now included in the table caption.

 

- Table 4. Column 1 - what does the *refer to on Mobile phone use proficiency*. 

 

Response: This has been reworded to “Mobile phone usage skills”.

 

- Figure 4. This figure does not substantively add to the paper. A brief description of the theory of behaviour change, and a reference would suffice.

 

Response: Thanks very much for the suggestion. However, we would like to keep the figure as we feel that it pictorially depicts what we wish to express better.

*Once again, we thank the reviewers immensely for their valuable comments. We hope we have addressed all the issues you have highlighted to your satisfaction.*

Best wishes,

*Authors*

# Peer review: Reviewer response for version 1

**DOI:** 10.21956/wellcomeopenres.16514.r35764

**Published:** 2019-07-23

**Authors:** Mimi Nichter

**Affiliations:** 1 School of Anthropology, University of Arizona, Tucson, AZ, USA

This article provides information on the potential for smoking cessation interventions in Indonesia. This is an important topic as Indonesia has one of the highest prevalence of smoking among men in the world and is a country where phone use is extensive. Although this exploratory study is focused on the acceptability and perceived usefulness of a mobile phone smoking cessation app, it fails to consider the importance of incorporating the cultural context of smoking and perceived harm of tobacco use into the intervention. We need to consider the extent to which an intervention developed in New Zealand or the UK would have equal relevance to the Indonesian smoker. While this is beyond the focus of the current study, it should be discussed as critically important for the success of any mHealth intervention developed for LMIC. 

1. Introduction: In relation to pharmaceuticals for nicotine addiction (patch, gum etc) - note that these medications, although available without prescription, are very expensive, and out of the realm of the possible for most Indonesians. (It would be interesting to know how much more they are than cigarettes, which are very inexpensive).
2. P. 4  Of the 850 visitors to the website, why did only 19% (n=161) complete the survey? This is a very low response rate. How long did the survey take to complete? Were respondents all from Java or from all over Indonesia? It is not clear from the discussion.
3. In one section, you mention 161 people as completing the survey, then in the socio-demographic data you note “Of the 123 respondents…” So how many people were there?
4. 83% of respondents were men; 17% were women—yet most studies to date show prevalence of women’s smoking in Indonesia is about 2-3%, so please explain this discrepancy.
5. In Table 1, provide IDR calculations to USD or to UK pounds so readers outside of Indonesia can interpret what the income figures mean.
6. Explain why only 75% of respondents were smokers. Why would the other 25% be interested in the intervention if they did not smoke? Were they ex-smokers who were concerned about relapse or people gathering information for family members?
7. Table 3: Do the authors have more detailed information on smoking status: daily smoker could be a very low level smoker (3 a day, or it could be 10 per day); occasional smoker could be defined differently by various people to mean once a week or twice a week or monthly, etc. The number of cigarettes smoked per day would be much more useful data, if available.
8. Consider eliminating the paragraph on what other uses the informants had for their phones (alarm function; texting, playing games...). It is not clear what relevance this has to the study at hand. Why is alarm use needed in Table 4? Why is the main use of mobile internet needed - it seems obvious that these are the features people want and use on a regular basis. If there is a reason for inclusion, please do make it clear.
9. Do people have to pay for text messages received in Indonesia or are they free, or does it depend on the plan? This might be important in people’s decision to want text messages.
10. p. 7. Describe what is meant by “permitted number of cigarettes for a day”. Who determines this? When is it determined? Is it in relation to number of cigarettes smoked per day prior to quitting?
11. Discussion, p. 7: the numbers cited in relation to DALYs are different than what is mentioned in introduction. Consider changing the first sentence to reflect the prevalence of smoking in Indonesia, and the lack of cessation services currently available.
12. With regards to knowledge about the harm of tobacco in Indonesia, the authors should review Padmawati, Ng *et al*. (2009) ^1^, in which it is noted that diabetes patients were believed to be able to smoke 3 cigarettes per day as relatively harmless while healthy people could smoke 12 cigarettes a day. See also Ng *et al.* (2010) ^2^. Also of use would be Nichter *et al*. (2009) ^3^, for details about perceptions of smoking and quitting in Java. It would be useful for the reader to understand a bit more about Indonesia and people’s attitudes toward quitting. A paragraph in the introduction would be sufficient and would highlight the reason for developing mobile phone apps for cessation and their potential in the country.
13. p. 7. Section on Quitting with mobile phones: Make it clear that at present behavior change interventions are not common in Indonesia; As your own data shows, few patients are getting a message to quit from their doctor.
14. Preferred mode of communication Figure 2: write out what SMS, MMS and IVR stand for.
15. Figure 2 & 3: Why does the n vary between figures?
16. Figure 3: Only a small percentage of your sample had smartphones, so what were they answering: what they would want in a smartphone if they had one?
17. Given that this study was done a few years ago, what is the prevalence of smartphone use now in Indonesia?
18. Explain early on what type of message would be delivered by SMS, MMS and IVR. How do they differ? Can you give examples from the intervention?
19. p. 10. It is unclear to the reader if any content for the proposed smoking cessation intervention has been developed or if the article is based solely on whether the audience would utilize an online intervention. Has thought been given or shared to the audience about the content of the intervention? Will it be based on an understanding of Indonesian smoker’s behaviors and ideas about quitting or will the intervention be a translation of a program normed and developed in another country? If so, which country? Are there plans for the smoking intervention to be adapted for the Indonesian cultural context? Please note that even high level smokers in Indonesia do not think of themselves as “addicted,”—which clearly has implications for thinking about quitting.
20. p. 10. The authors state that few studies have explored use of smartphones for interventions. Hasn’t the CDC in the US developed a large smoking cessation intervention? This is not a new phenomena. Further on p. 11, you review many of these interventions, so these sections need to be combined.
21. p. 10  The information about smartphone apps for a smoking cessation intervention should be moved into the introduction, as well as info on an MMS-based intervention. All of this information about development of apps in other countries should be noted in the beginning of the article so the reader understands a bit of the context.
22. p. 11 Move the information about the UK to the introduction. You should also note that messages to avoid environments where people smoke are extremely problematic in Indonesia where over 65% of men smoke and where few smoke free restrictions are in place.
23. p. 11 “the profound ethnic diversity of Indonesia…if considered..might increase the complexity and cost of the intervention”…Yes surely it would, but utilizing a ‘one size fits all’ approach does not seem like a recipe for success.
24. p. 12.  Methodological Issues: Perhaps rename this as Limitations.The authors note the levels of tobacco dependency were low among respondents. It is possible that the reason for this is that the Fagerstrom as a measure of nicotine dependence in Indonesia is not very robust because smoking patterns are very different. This has been addressed in several publications of the Quit Tobacco International Project, conducted in Yogyakarta, the site of the present study.
25. p. 12 Conclusion: reword line one to indicate “Our study showed that among those smokers in Indonesia who responded to our survey…” rather than “our study showed that people in Indonesia who smoke (which seems a bold claim for a small survey).

Is the work clearly and accurately presented and does it cite the current literature?

Partly

If applicable, is the statistical analysis and its interpretation appropriate?

Yes

Are all the source data underlying the results available to ensure full reproducibility?

No source data required

Is the study design appropriate and is the work technically sound?

Partly

Are the conclusions drawn adequately supported by the results?

Partly

Are sufficient details of methods and analysis provided to allow replication by others?

Partly

Reviewer Expertise:

I am a medical anthropologist who has conducted extensive research on the development of tobacco cessation programs in Java, Indonesia.

I confirm that I have read this submission and believe that I have an appropriate level of expertise to confirm that it is of an acceptable scientific standard, however I have significant reservations, as outlined above.

## References

[1] : Smoking among diabetes patients in Yogyakarta, Indonesia: cessation efforts are urgently needed. *Trop Med Int Health*.2009;14(4) : 10.1111/j.1365-3156.2009.02241.x 412-9 10.1111/j.1365-3156.2009.02241.x 19228351

[2] : Bringing smoking cessation to diabetes clinics in Indonesia. *Chronic Illn*.2010;6(2) : 10.1177/1742395310364253 125-35 10.1177/1742395310364253 20444767

[3] : Anthropological contributions to the development of culturally appropriate tobacco cessation programs. *Anthropology and Public Health: Bridging Differences in Culture and Society*.2009;

# Response

**Published:** 2020-08-07

**Authors:** Rashmi Rodrigues

**Affiliations:** St John's National Academy of Health Sciences, India

Dear Dr. Nichter,

 We very much appreciate and thank you for your valuable time on reviewing our work. We have addressed your comments to the best of our ability and sincerely hope we have done justice to your suggestions on the report. The changes have been mentioned in a point-wise format as mentioned below, and a second version of the manuscript has been submitted to the journal.

 We have added information on the sampling strategy, rephrased the table titles and removed associations for which adjusted odds ratios were unavailable among many other minor corrections, in line with the feedback received.

 We hope the revised manuscript meets the standards to be considered for a formally approved status on Wellcome Open Research.

 Thanking you again,

 Sincerely,

 Mochammad Fadjar Wibowo

 Submitting Author

 (on behalf of all the authors)

**RESPONSE TO REVIEWERS:**

 EXTERNAL PEER-REVIEWER #1 (Mimi Nichter, PhD)

 Thank you once again for your comments. We have taken note of your detailed feedback on various sections in the manuscript and have addressed them to the best of our ability. Please find our point-counterpoint responses to your comments below:

  

- Introduction: In relation to pharmaceuticals for nicotine addiction (patch, gum etc) - note that these medications, although available without prescription, are very expensive, and out of the realm of the possible for most Indonesians. (It would be interesting to know how much more they are than cigarettes, which are very inexpensive).

We have added this information under the ‘Introduction’ section paragraph 2 per your comment.

 In Indonesia, nicotine replacement therapy such as nicotine patches, gums, and sprays are available without prescription in pharmacies but cost 7000-250,00 IDR (0.0035-0.035 USD) per unit. Quitting with prescription medication such as varenicline, hypnotherapy or  behavioural therapy cost 0.8-1million IDR and are not very popular ^6^ . Further, most (71%) of those who want to quit smoking, attempt to quit without assistance while others may use traditional methods (herbal or medicinal plants), smokeless tobacco or counselling ^6^ . The costs of quitting vs the costs of cigarettes (12-24000 IDR/ pack currently and 32-53000 IDR from 1 ^st^ November 2019) probably influences both the quit strategy and the decision to continue smoking. Additionally, the self-confidence of Indonesian physicians in providing smoking cessation counselling is reportedly low, with smoking cessation services offered only at a few healthcare facilities ^8^ . There is also no national toll-free quit-line ^9^ . This prevailing scenario makes innovation and improvement in smoking cessation interventions in Indonesia a necessity.

  

- P. 4  Of the 850 visitors to the website, why did only 19% (n=161) complete the survey? This is a very low response rate. How long did the survey take to complete? Were respondents all from Java or from all over Indonesia? It is not clear from the discussion.

**Answer:**

 Participants took 34:06 (±2:02) minutes to complete the questionnaire. – this is now mentioned in the *last sentence of the 5 ^th^ paragraph in the methods section* of the manuscript.

 The respondents’ locations represented 14 of the 34 provinces in Indonesia- this was already mentioned in the *1 ^st^ paragraph of the results section* of the manuscript.

  

- In one section, you mention 161 people as completing the survey, then in the socio-demographic data you note “Of the 123 respondents…” So how many people were there?

While 161 respondents completed the questionnaire, not all responded to every single question. Also, the questionnaire was designed such that respondents could skip questions based on their responses to previous questions, which changed the ‘n’ for various sections and questions of the questionnaire.

- 83% of respondents were men; 17% were women—yet most studies to date show prevalence of women’s smoking in Indonesia is about 2-3%, so please explain this discrepancy.

While there were only 124 of the 161 respondents who provided complete demographic information, especially information on gender, we believe that the difference may be due to internet accessibility and receipt of information regarding the survey. Unlike the prevalence of smoking, the distribution of men and women accessing the internet and using social media (using which the questionnaire was made available) in Indonesia is approximately 50% each and the gender distribution in the study is a consequence of internet usage patterns.

- In Table 1, provide IDR calculations to USD or to UK pounds so readers outside of Indonesia can interpret what the income figures mean.

USD to IDR conversions have been added to Table 1, 2 and 4.

  

- Explain why only 75% of respondents were smokers. Why would the other 25% be interested in the intervention if they did not smoke? Were they ex-smokers who were concerned about relapse or people gathering information for family members?

**Answer:**

 The questionnaire was administered to respondents to ever smoked. While 75% were current smokers, ex-smokers were required to respond to smokers ‘hypothetically’ in a scenario of the mobile phone intervention being was made available when they were attempting to quit. We believe that they would be able to use their quitting experience to better advise us regarding an intervention that enabled quitting. Unfortunately, we did not obtain information on whether they were concerned about relapse.

 We have mentioned this in the *5 ^th^ paragraph* of the methods section of the manuscript as follows:

*‘These respondents were current and former smokers, aged 18 years or older, residing in Indonesia for the past year.’*


*  *

- Table 3: Do the authors have more detailed information on smoking status: daily smoker could be a very low level smoker (3 a day, or it could be 10 per day); occasional smoker could be defined differently by various people to mean once a week or twice a week or monthly, etc. The number of cigarettes smoked per day would be much more useful data, if available.

We do agree with the reviewer that the number of cigarettes smoked would be more useful data.

 The smoking status used in the analysis was obtained from the question i.e., the response to the question Do you currently smoke tobacco on a daily basis, less than daily, or not at all?

 However, we also requested participants to report the number of cigarettes smoked/ day. Unfortunately, only 100 reported the number of cigarettes smoked/ day or week. Participants reported smoking an average 9(SD8) cigarettes/ day, ranging from <1-32 cigarettes/ day. Hence, we used their self-report of smoking daily or occasionally or in the past as an indicator of their current smoking status. Most participants had smoked 3 pack years and ranged from 0.1-41 pack years. For the purpose of calculating pack years we assumed cigarette packs despite the different types of tobacco smoked.

 We have now also reported the number of cigarettes smoked in a day in the results section under ***Smoking status characteristics.***

- Consider eliminating the paragraph on what other uses the informants had for their phones (alarm function; texting, playing games...). It is not clear what relevance this has to the study at hand. Why is alarm use needed in Table 4? Why is the main use of mobile internet needed - it seems obvious that these are the features people want and use on a regular basis. If there is a reason for inclusion, please do make it clear.

Thank you for the suggestion - however, we would like to keep the paragraph as it suggests the other features that could be incorporated into the design of a mobile phone application for smoking cessation.

- Do people have to pay for text messages received in Indonesia or are they free, or does it depend on the plan? This might be important in people’s decision to want text messages.

Receiving text messages in Indonesia is free irrespective of the plan. It however costs IDR 360 (3 cents) to send a message, while bulk messaging would cost upto IDR 65,000 (USD 4.5) for 500 messages a month. These costs would be incurred by the intervention provider.

  

- p. 7. Describe what is meant by “permitted number of cigarettes for a day”. Who determines this? When is it determined? Is it in relation to number of cigarettes smoked per day prior to quitting?

**Answer:**

 We have revised this sentence to read- *‘reminders about the numbers of cigarettes that they could smoke each day as they approached their quit date’*

 Studies have indicated that reduction in smoking is easier to achieve than complete cessation and once achieved could promote complete cessation. Reduction reverses neuroadaptation to tobacco and minimises the symptoms of withdrawal and the cravings experienced with sudden complete cessation, therefore a target is set each day in terms of minimising the cigarettes smoked until the quit date is reached.

*West RJ et al Psychol Med. 1989 Nov; 19(4):981-5.*

  

- Discussion, p. 7: the numbers cited in relation to DALYs are different than what is mentioned in introduction. Consider changing the first sentence to reflect the prevalence of smoking in Indonesia, and the lack of cessation services currently available.

**Answer:**

 Thank you very much for highlighting this. The discussion however indicated global DALYs while the introduction indicated the DALYs lost in Indonesia due to smoking.

 However, in the interest of minimizing confusion we have rewritten the first paragraph of the discussion to read as follows:

 In Indonesia, an LMIC, the burden of tobacco smoking has risen from 59 million in 2000 to 70 million in 2010 and 73.6 million in 2015 and continues to rise, so does the amount spent on tobacco related illnesses. The situation is compounded by the limited awareness regarding the hazards of smoking along with the minimalistic support available to quit. Finding innovative solutions that are acceptable to Indonesians wanting to quit is therefore essential. Therefore, given the current pervasiveness of mobile phone communication and its minimal cost we sought to explore the acceptability and design for a mobile phone smoking cessation intervention in Indonesia.

- With regards to knowledge about the harm of tobacco in Indonesia, the authors should review Padmawati, Ng et al. (2009), in which it is noted that diabetes patients were believed to be able to smoke 3 cigarettes per day as relatively harmless while healthy people could smoke 12 cigarettes a day. See also Ng et al. (2010). Also, of use would be Nichter et al. (2009), for details about perceptions of smoking and quitting in Java. It would be useful for the reader to understand a bit more about Indonesia and people’s attitudes toward quitting. A paragraph in the introduction would be sufficient and would highlight the reason for developing mobile phone apps for cessation and their potential in the country.

We agree with your thoughts on the baseline understanding of smoking and its effects among Indonesian subjects and have added a paragraph highlighting perceptions under the ‘Introduction’ section as follows:

 Further, there are varied perceptions of the effects and complications of tobacco on health in Indonesia. For example; some patients with diabetes mellitus considered that they could smoke relatively lesser cigarettes/ day (3/day) when compared with those who were healthy (12/ day) *[Insert Ref D],* while others it did not know it could complicate their illness *[Insert Ref E]*. Also, reducing or quitting smoking was considered an option only for those seriously ill and could be resumed on recovery *.* *[Insert Ref D]*. This prevailing scenario makes innovation and improvement in smoking cessation interventions in Indonesia a necessity.

- p. 7. Section on Quitting with mobile phones: Make it clear that at present behavior change interventions are not common in Indonesia; As your own data shows, few patients are getting a message to quit from their doctor.

Answer:

 Thank you once again for the suggestion. We have made the change and introduced the following paragraph under the section on *‘Quitting with mobile phones’*

 Currently in Indonesia behaviour change interventions are uncommon and when available are expensive. So also, is advise from physicians regarding quitting. Our study indicated that nearly half the participants did not receive any advice from their healthcare provider to quit, despite a reported desire to do so.

- Preferred mode of communication Figure 2: write out what SMS, MMS and IVR stand for.

Thank you for comment we have now expanded these abbreviations. 

- Figure 2 & 3: Why does the n vary between figures?

We acknowledge that discrepancies in the numbers (n) between variables exist, these are due to missing data especially demographic, as respondents most probably chose not to respond to them. We have now discussed the discrepancy in the ‘limitations section’ of the manuscript.

- Figure 3: Only a small percentage of your sample had smartphones, so what were they answering: what they would want in a smartphone if they had one?

In the “Mobile phone usage patterns” section under “Results”, we had stated that 154 (98%) of our respondents used a smartphone which reflects a majority. The minority of subjects who did not own a smartphone (7, 2%) answered the questions on desired features of an application had they owned a smartphone as you mentioned in your comment. We have thus made no changes here.

- Given that this study was done a few years ago, what is the prevalence of smartphone use now in Indonesia?

We have described the trend in mobile phone usage in Indonesia under the section ‘ *Mobile phone penetration and mHealth development in Indonesia’ *of the introduction as follows:

 The growth of mobile users in Indonesia is one of the fastest in Asia with a steady increase from 125 per 100 people in 2013 to 173 per 100 people in 2018 ^20^ . Given the improving internet accessibility and low cost of smartphones ^21^ , with prices as low as 40 USD for a phone, smartphone penetration in Indonesia has reached 27% in 2018 ^22^ and is predicted to reach 32% by 2022 ^23^ .

- Explain early on what type of message would be delivered by SMS, MMS and IVR. How do they differ? Can you give examples from the intervention?

**Answer:**

 Thank you for the suggestion: we have now explained this in the introduction (7 ^th^ paragraph) as follows:

  Short Messaging Service (SMS) Multimedia Messaging Service (MMS) (pictoral messages), live-voice calls and interactive voice response (IVR) technology that replace a human caller with a computer, provide motivation and counseling to those who want to quit smoking ^11^ . Such messages use prompts (either text i.e.; SMS, picture i.e, MMS or voice i.e., calls and IVR) to encourage avoiding cigarettes, ashtrays, lighters, and environments where people usually smoke eg.; ‘For the next 4 hours, stay away from cigs’. Additionally, messages also help identify the challenges to quitting and the plan to overcome them. Prompts to use telephone helplines and nicotine replacement therapy ^17^ , economic savings from quitting and nutritional advise form the content of messages used to support quitting.

- p. 10. It is unclear to the reader if any content for the proposed smoking cessation intervention has been developed or if the article is based solely on whether the audience would utilize an online intervention. Has thought been given or shared to the audience about the content of the intervention? Will it be based on an understanding of Indonesian smoker’s behaviors and ideas about quitting or will the intervention be a translation of a program normed and developed in another country? If so, which country? Are there plans for the smoking intervention to be adapted for the Indonesian cultural context? Please note that even high level smokers in Indonesia do not think of themselves as “addicted,”—which clearly has implications for thinking about quitting.

We had not developed any content for the intervention when the study was initiated. Based on this study and available funding support further development of the intervention will happen for which intensive work into what content and its wording is acceptable to the Indonesian population will be identified and used. In this study we have aimed only to identify the kind of communication that is acceptable, its frequency and some basic content.

- p. 10. The authors state that few studies have explored use of smartphones for interventions. Hasn’t the CDC in the US developed a large smoking cessation intervention? This is not a new phenomena. Further on p. 11, you review many of these interventions, so these sections need to be combined.

Thank you for highlighting this. Our aim was to indicate that such interventions have been used minimally in low-middle income contexts such as Indonesia. However, based on your suggestion we think it best to remove the paragraph from the manuscript. We however choose keep the sections as they are. 

- p. 10  The information about smartphone apps for a smoking cessation intervention should be moved into the introduction, as well as info on an MMS-based intervention. All of this information about development of apps in other countries should be noted in the beginning of the article so the reader understands a bit of the context.

**Answer:** We have moved part of the content to the introduction as indicated in response 18

- p. 11 Move the information about the UK to the introduction. You should also note that messages to avoid environments where people smoke are extremely problematic in Indonesia where over 65% of men smoke and where few smoke free restrictions are in place.

We have moved the information on UK to the introduction as best as we can. We understand that messages to avoid environments where people smoke are extremely problematic in Indonesia where over 65% of men smoke. We will ensure that the intervention designed (based on available funding) will have an extensive situational analysis prior to design and deployment.

- p. 11 “the profound ethnic diversity of Indonesia…if considered..might increase the complexity and cost of the intervention”…Yes surely it would, but utilizing a ‘one size fits all’ approach does not seem like a recipe for success.

Thank you. We do not recommend a one size fits all approach. What we suggest is a menu of features within an application, identified based on potential user surveys. The beneficiaries/ users and then choose the features that best suit them. However, this will make the intervention complex and difficult to test scientifically.

- p. 12.  Methodological Issues: Perhaps rename this as Limitations. The authors note the levels of tobacco dependency were low among respondents. It is possible that the reason for this is that the Fagerstrom as a measure of nicotine dependence in Indonesia is not very robust because smoking patterns are very different. This has been addressed in several publications of the Quit Tobacco International Project, conducted in Yogyakarta, the site of the present study.

Thank you!

 We have renamed methodological issues as *‘limitations’*

 We have included your suggestion in the limitations as follows:

 However, the level of nicotine dependence obtained may be questionable given the normalization of smoking in Indonesia.  Further, social desirability bias also cannot be ruled out in the FTND as it is a self-report of dependence by the participant.

- p. 12 Conclusion: reword line one to indicate “Our study showed that among those smokers in Indonesia who responded to our survey…” rather than “our study showed that people in Indonesia who smoke (which seems a bold claim for a small survey).

This sentence has been reworded to reflect more clarity per your suggestion as follows:

 Our study showed that smokers in Indonesia who responded to our survey perceived receiving a potential smoking cessation intervention via mobile phones as useful.

*Once again, we thank the reviewers immensely for their valuable comments. We hope we have addressed all the issues you have highlighted to your satisfaction.*

 Best wishes,

*Authors*
